# Evaluation of Environmental Impacts Due to Blue Water Consumption in China from Production and Consumption Perspectives

**DOI:** 10.3390/ijerph15112445

**Published:** 2018-11-02

**Authors:** Jing Liu, Mengyang Wu, Zhongbo Yu

**Affiliations:** 1 State Key Laboratory of Hydrology-Water Resources and Hydraulic Engineering, Hohai University, Nanjing 210098, Jiangsu, China; liujing0027@hhu.edu.cn; 2 Joint International Research Laboratory of Global Change and Water Cycle, Hohai University, Nanjing 210098, Jiangsu, China; 3 College of Hydrology and Water Resources, Hohai University, Nanjing 210098, Jiangsu, China; 4 College of Agricultural Engineering, Hohai University, Nanjing 210098, Jiangsu, China; wumengyang_hehai@126.com

**Keywords:** blue water consumption, environmental impacts, water footprint, China, production, consumption

## Abstract

Current environmental impact analyses are mainly focused on land, soil, energy, and material consumption, while studies regarding blue water consumption are scarce. Based on the water footprint concept, this study evaluates the impacts of blue water consumption on human health, ecosystem quality, and water resources in China from the production and consumption perspective, respectively. The results indicate that environmental impacts due to blue water consumption in China were 15.82 × 10^6^ DALY (disability-adjusted life years), 96.54 × 10^9^ m^2^∙year, and 175.20 × 10^9^ MJ, and provinces such as Xinjiang, Shandong, and Hebei could be targets for achieving smaller environmental impacts in the future. More than 80% of environmental impacts were related to the agricultural sector. In terms of agricultural production, about 70% of the environmental impacts were related to product export. Measures such as the shift of the agricultural production pattern from water-intensive crops and animal products toward less water-intensive ones, the increase of agricultural water use efficiency, and the adoption of water-saving technologies could contribute to smaller environmental impacts. In terms of agricultural consumption, more than 95% of the environmental impacts were related to agricultural products produced locally. The focus was on increasing awareness of the importance of saving water and whether products were imported from regions with relatively small environmental impacts.

## 1. Introduction

Water is one of the most important resources in the world, and how to keep water consumption at a sustainable level has become more and more difficult due to the growing population and changing climate, among other factors [1,2,3]. Compared with green water, the opportunity costs and environmental influences of blue water are more significant, and blue water use in irrigated agriculture has the potential to cause severe environmental problems such as water depletion, salinization, waterlogging, or soil degradation [4,5,6,7]. Consequently, a number of research works focused on blue water use have been done. Based on the study of Kummu et al., the global blue water consumption per capita has increased from 209 m^3^/(year∙capita) in the 1900s to 230 m^3^/(year∙capita) in the 2000s, and total blue water consumption increased fourfold within the same period when taking the increase of the global population into consideration [2,8]. The blue water uses in America [9,10], China [11], Cyprus [12], and the United Kingdom [13] have also been evaluated and different sectors including agricultural, industrial, and domestic sectors have been included. Compared with the traditional or restricted measure of water withdrawal, the water footprint could be regarded as a comprehensive freshwater appropriation indicator, which could illustrate the volume of blue water used to produce products over the full supply chain from different perspectives [14]. Ercin and Hoekstra estimated the blue water footprint of Europe in 2050 at the country level from both production and consumption perspectives and analyzed the main drivers [15]. Their study illustrated that both the water footprint of production and the water footprint of consumption were significantly influenced by consumption pattern. At the irrigation district scale, the blue water consumption in the agricultural, industrial, and domestic sectors was also evaluated from the production and consumption perspectives, respectively, and the corresponding blue water scarcity was also analyzed [16,17]. Besides the production and consumption perspectives, the internal and external water footprints for each country of the world were calculated by Hoekstra and Chapagain [18], and Hoekstra and Mekonnen found that about half of the United Kingdom’s blue water footprint was located in regions where the blue water footprint exceeded a sustainable level [19]. The environmental impact analysis is one of the most important research fields, and many studies on the environmental impacts of land and soil use, energy use, and material consumption have been conducted [2,20,21]. However, the environmental analyses for blue water consumption, especially those based on the indicator of the water footprint, are scarce [22,23,24].

Minimizing environmental impacts is one of the challenges we are facing in the 21st century [25,26]. Based on the concept of water footprint, this study evaluates the environmental impacts (the impacts on human health, ecosystem quality, and water resources) due to blue water consumption in China from the production and consumption perspectives respectively, This study could feed the discussion about sustainable water consumption and also forms a good basis for local water management.

## 2. Materials and Methods

### 2.1. Methods

#### 2.1.1. Water Footprint of Production and Water Footprint of Consumption

Virtual water, as it is known, is the water embedded in commodities [27] or the water required for the production of commodities [28]. In this study, virtual water content means the water required for the production of commodities per unit of mass (m^3^/kg) [29]. The values of virtual water content for crops and those for animal products were taken from previous studies [30,31], where the former was calculated based on a grid-based dynamic water balance model and CROPWAT 8.0 model and the latter was focused on water consumption related to animal feed and drinking water and service [32,33]. Thus, the water footprint of production for the agricultural sector could be calculated by multiplying a product’s virtual water content by the product’s output, and the water footprint of consumption for the agricultural sector could be calculated by multiplying the product’s virtual water content by the product’s consumption. In this study, seven kinds of crops (cereals, beans, tubers, oil-bearing crops, sugar crops, vegetables, and fruits) and six kinds of animal products (pork, beef, mutton, poultry, milk, and eggs) were included.

From the production perspective, products produced locally could either be used by the local inhabitants or be exported to other areas to meet the needs of inhabitants there. From the consumption perspective, products consumed locally could either be met by the local production or be imported from other areas. The exporting of products means more water consumption, and the importing of products could be seen as a component of water availability based on the fact that water consumed in imported products enables the saving of local water for other purposes [17,34,35,36]. We assumed that the product trading was based on the surpluses and deficits method which has been used in previous studies [6,17,36]. Then, the two parts for the water footprint of agricultural production (for products used locally and exported to other areas) and the two parts for the water footprint of agricultural consumption (from local production and imported products) could be obtained.

For the industrial sector, domestic sector, and artificial ecosystem, the water footprint of production could be calculated by multiplying water withdrawal by the related consumption ratio [17,37,38,39]. The water footprints of consumption for these sectors were not studied in this work based on data availability.

#### 2.1.2. Impacts on Human Health, Ecosystem Equality and Resources

The method we used in this study was based on the framework of the Eco-indicator-99 assessment methodology [40], which was a damage-oriented method for life cycle impact assessment, and this method could also be adapted to similar life cycle impact assessment methods, such as LIME [41] or IMPACT 2002+ [42]. Competition in water-scarce regions could affect irrigation, which might result in malnutrition. In developing countries, water shortage is usually associated with numerous influencing factors, such as physical water scarcity, and socioeconomic parameters are also relevant for mitigation measures of potential health damage [43,44]. Thus, the impacts of water consumption on human health in this study were calculated as follows, considering the cause-effect chain [44]:

(1) $${\mathrm{HH}}_{\mathrm{pro}}={\mathrm{WSI}}^{\mathrm{loc}}\cdot {\mathrm{WU}}_{\%,\mathrm{agr}}^{\mathrm{loc}}\cdot {\mathrm{HDF}}^{\mathrm{loc}}\cdot \frac{1}{{\mathrm{WR}}^{\mathrm{loc}}}\cdot {\mathrm{DF}}^{\mathrm{loc}}\cdot {\mathrm{WF}}_{\mathrm{pro}}$$

(2) $$\begin{array}{cc} {\mathrm{HH}}_{\mathrm{con}} & ={\mathrm{WSI}}^{\mathrm{loc}}\cdot {\mathrm{WU}}_{\%,\mathrm{agr}}^{\mathrm{loc}}\cdot {\mathrm{HDF}}^{\mathrm{loc}}\cdot \frac{1}{{\mathrm{WR}}^{\mathrm{loc}}}\cdot {\mathrm{DF}}^{\mathrm{loc}}\cdot {\mathrm{WF}}_{\mathrm{con}-\mathrm{loc}}\\ & +{\mathrm{WSI}}^{\mathrm{imp}}\cdot {\mathrm{WU}}_{\%,\mathrm{agr}}^{\mathrm{imp}}\cdot {\mathrm{HDF}}^{\mathrm{imp}}\cdot \frac{1}{{\mathrm{WR}}^{\mathrm{imp}}}\cdot {\mathrm{DF}}^{\mathrm{imp}}\cdot {\mathrm{WF}}_{\mathrm{con}-\mathrm{imp}} \end{array}$$

where ${\mathrm{HH}}_{\mathrm{pro}}$ ${\mathrm{HH}}_{\mathrm{con}}$ are the impacts of water consumption on human health due to blue water consumption related to production and consumption, respectively, measured in disability-adjusted life years (DALY); ${\mathrm{WSI}}^{\mathrm{loc}}$ and ${\mathrm{WSI}}^{\mathrm{imp}}$ are the physical water stress indexes at the producing and importing regions, respectively, which were based on the study of Pfister et al. [44]; ${\mathrm{WU}}_{\%,\mathrm{agr}}^{\mathrm{loc}}$ and ${\mathrm{WU}}_{\%,\mathrm{agr}}^{\mathrm{imp}}$ are the percentages of agricultural water use at the producing and importing regions, respectively; ${\mathrm{HDF}}^{\mathrm{loc}}$ and ${\mathrm{HDF}}^{\mathrm{imp}}$ are the human development factors at the producing and importing regions, respectively, which could reflect the influences of the human development index on malnutrition vulnerability, and they were calculated using Equation (2); ${\mathrm{WR}}^{\mathrm{loc}}$ and ${\mathrm{WR}}^{\mathrm{imp}}$ are the per-capita water requirements to prevent malnutrition at the producing and importing regions, respectively (m^3^/(year·capita)), and they were assumed as 1350 m^3^/(year·capita), which was the minimum direct human dietary requirement; ${\mathrm{DF}}^{\mathrm{loc}}$ and ${\mathrm{DF}}^{\mathrm{imp}}$ are the damage factor at the producing and importing regions, respectively (DALY/(year·capita)), which could show the damage caused by malnutrition, and they were assumed as 0.0184 DALY/(year·capita), which was derived on a country level from linear regression of the malnutrition rate and DALY value for malnutrition per 100,000 people in 2002 [44,45,46]; and ${\mathrm{WF}}_{\mathrm{pro}}$, ${\mathrm{WF}}_{\mathrm{con}-\mathrm{loc}}$, and ${\mathrm{WF}}_{\mathrm{con}-\mathrm{imp}}$ are the volumes of the water footprint related to production, consumption from local products, and consumption from imported products (m^3^), respectively.

(3) $$\mathrm{HDF}=\left\{ \begin{array}{lll} 1 & & \left(\mathrm{HDI}<0.30\right)\\ 2.03{\mathrm{HDI}}^{2}-4.09\mathrm{HDI}+2.04 & & \left(0.30\leq \mathrm{HDI}\leq 0.88\right)\\ 0 & & \left(\mathrm{HDI}>0.88\right) \end{array}\right.$$

where HDI is the human development index.

Water consumption means less water availability and it has a negative influence on regional biodiversity, which contributes significantly to regional ecosystem quality. Especially in semiarid and arid regions, terrestrial ecosystems are usually runoff-dependent, and biodiversity in these regions contributes significantly to ecosystem quality within a watershed [44,47,48]. In this study, the impacts of water consumption on ecosystem quality related to the water footprint of production (${\mathrm{EQ}}_{\mathrm{pro}}$ (m^2^·year)) and the water footprint of consumption (${\mathrm{EQ}}_{\mathrm{con}}$ (m^2^·year)) are calculated as follows [40,44]:

(4) $${\mathrm{EQ}}_{\mathrm{pro}}={\mathrm{IF}}^{\mathrm{loc}}\cdot {\mathrm{WF}}_{\mathrm{pro}}$$

(5) $${\mathrm{EQ}}_{\mathrm{con}}={\mathrm{IF}}^{\mathrm{loc}}\cdot {\mathrm{WF}}_{\mathrm{con}-\mathrm{loc}}+{\mathrm{IF}}^{\mathrm{imp}}\cdot {\mathrm{WF}}_{\mathrm{con}-\mathrm{imp}}$$

where ${\mathrm{IF}}^{\mathrm{loc}}$ and ${\mathrm{IF}}^{\mathrm{imp}}$ are the ecosystem quality-influencing factors at the producing and importing regions, respectively (m^2^·year/m^3^). The fraction of net primary production limited by water availability represents the water-shortage vulnerability of an ecosystem. They were calculated by aggregating these fractions by each region, using regionally specific precipitation as a weighting factor [44].

Water resource depletion could be caused by the extraction of fossil groundwater or the overuse of other water bodies [44]. To evaluate the impacts of water consumption on regional resources related to the water footprint of production ($R_{\mathrm{pro}}$ (MJ)) and to the water footprint of consumption ($R_{\mathrm{con}}$ (MJ)), the concept of backup technology (the surplus energy to make resources available in the future) was used in this study [49], and seawater desalination was used as the backup technology in China [44,49]. Thus, the impacts of water consumption on resources could be calculated as follows:

(6) $$R_{\mathrm{pro}}=E_{\mathrm{des}}^{\mathrm{loc}}\cdot F_{\mathrm{dep}}^{\mathrm{loc}}\cdot {\mathrm{WF}}_{\mathrm{pro}}$$

(7) $$R_{\mathrm{con}}=E_{\mathrm{des}}^{\mathrm{loc}}\cdot F_{\mathrm{dep}}^{\mathrm{loc}}\cdot {\mathrm{WF}}_{\mathrm{con}-\mathrm{loc}}+E_{\mathrm{des}}^{\mathrm{imp}}\cdot F_{\mathrm{dep}}^{\mathrm{imp}}\cdot {\mathrm{WF}}_{\mathrm{con}-\mathrm{imp}}$$

where $E_{\mathrm{des}}^{\mathrm{loc}}$ and $E_{\mathrm{des}}^{\mathrm{imp}}$ are the energy required for seawater desalination at the producing and importing regions, respectively (MJ/m^3^), and they were assumed as 11 MJ/m^3^ according to the state-of-the-art energy demand [50]; $F_{\mathrm{dep}}^{\mathrm{loc}}$ and $F_{\mathrm{dep}}^{\mathrm{imp}}$ are the characterization factors for freshwater depletion at the producing and importing regions, respectively, and they were calculated as follows [44]:

(8) $$F_{\mathrm{dep}}=\left\{ \begin{array}{lllll} 1-\frac{\mathrm{WA}}{\mathrm{WU}} & & & & \left(\mathrm{WU}>\mathrm{WA}\right)\\ 0 & & & & \left(\mathrm{WU}\leq \mathrm{WA}\right) \end{array}\right.$$

where WU is water withdrawal (m^3^) and WA is local freshwater availability (m^3^). If water withdrawal was smaller than water availability, the water depletion would not exist due to the precipitation compensation; otherwise, water depletion could occur.

### 2.2. Data Sources

Crop and animal products’ output and consumption and human population were from the Statistical Yearbook of China and Agricultural Statistical Data of China [51,52]. Data on water withdrawals of different sectors, water availability, and water consumption ratio were from the China Water Resources Bulletin, and HDI was from the China National Human Development Report [53,54].

## 3. Results

### 3.1. Impacts on Human Health

**Figure 1:**
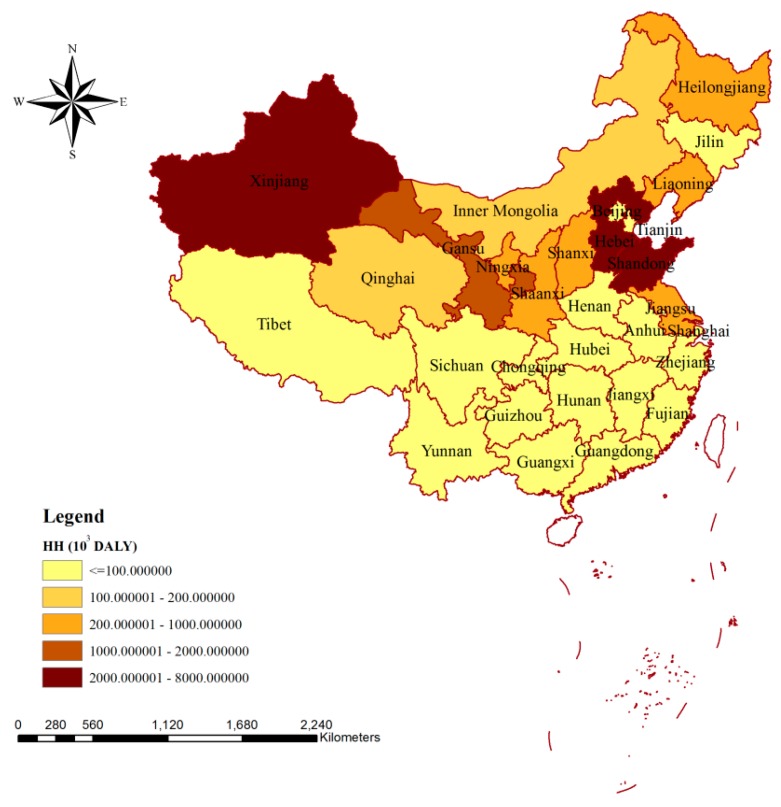
Spatial variation of the impacts of blue water consumption on human health via production in China. Note: HH means the impacts on human health caused by production.

Figure 1 shows the spatial variation of the impacts on human health due to the blue water consumption during production in China, and obvious differences can be observed. For Xinjiang, Hebei, and Shandong, the impacts on human health were more significant than other provinces, with the values of 6.87 × 10^6^, 2.67 × 10^6^, and 2.15 × 10^6^ DALY, respectively. For the rest of the provinces, Gansu was the only place where the influence of blue water consumption during production was more than 1.00 × 10^6^ DALY. There were 18 provinces in which human health was influenced by blue water consumption by less than 100 × 10^3^ DALY, and the smallest influence was found in Tibet (2.75 × 10^3^ DALY).

**Figure 2:**
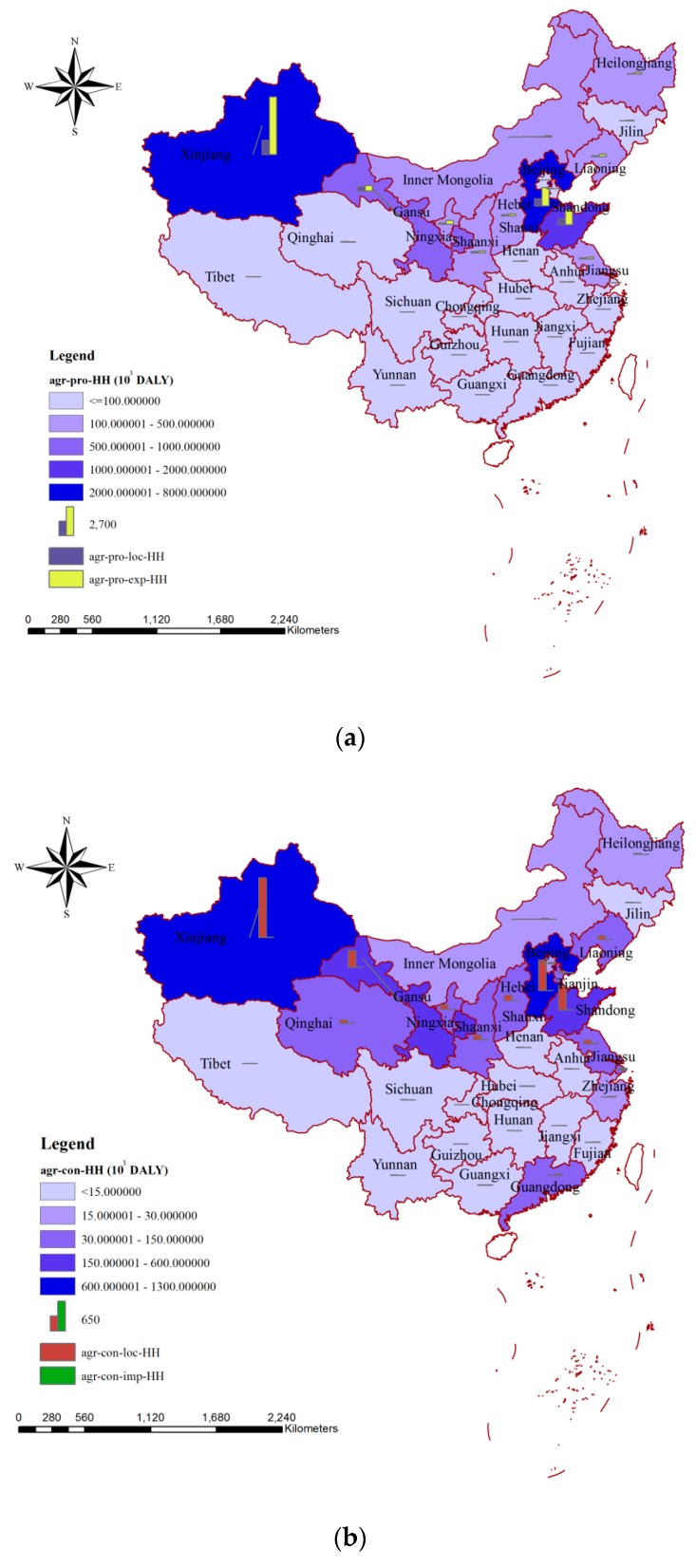
Spatial variation of the impacts of blue water consumption on human health via agricultural production (**a**) and agricultural consumption (**b**) in China. Note: agr-pro-HH, agr-pro-loc-HH, and agr-pro-exp-HH mean the impacts on human health due to agricultural production, the part of agricultural production used for local consumption, and the part of agricultural production exported to meet the needs of inhabitants in other areas, respectively. agr-con-HH, agr-con-loc-HH, and agr-con-imp-HH mean the impacts on human health due to agricultural consumption, the part of agricultural consumption met by local production, and the part of agricultural consumption dependent on imports from other areas, respectively.

As the sector of largest blue water consumption, the impacts of blue water consumption on human health from agricultural production and consumption perspectives are shown in Figure 2a,b, respectively. Similar to Figure 1, Xinjiang, Hebei, and Shandong occupied the top three places, both from the agricultural production and consumption perspectives (Figure 2). For the impacts of blue water consumption on human health via agricultural production, the smallest value could be seen in Shanghai (1.76 × 10^3^ DALY), while for the impacts due to agricultural consumption, the smallest one was provided by Tibet (1.59 × 10^3^ DALY). The blue water consumed by agricultural production could either be used for local consumption or be exported to other areas to meet the needs of inhabitants there. There were 20 provinces for which the impacts of blue water consumption on human health via agricultural production were mainly related to exported agricultural products (Figure 2a). The blue water consumed due to agricultural product consumption could either be met by local production or be imported from other areas. In China, the impacts of blue water consumption on human health via agricultural product consumption were mainly due to the contribution from local production for most of the provinces, excluding Fujian, Chongqing, Zhejiang, Guangdong, Beijing, and Shanghai (Figure 2b).

### 3.2. Impacts on Ecosystem Quality

**Figure 3:**
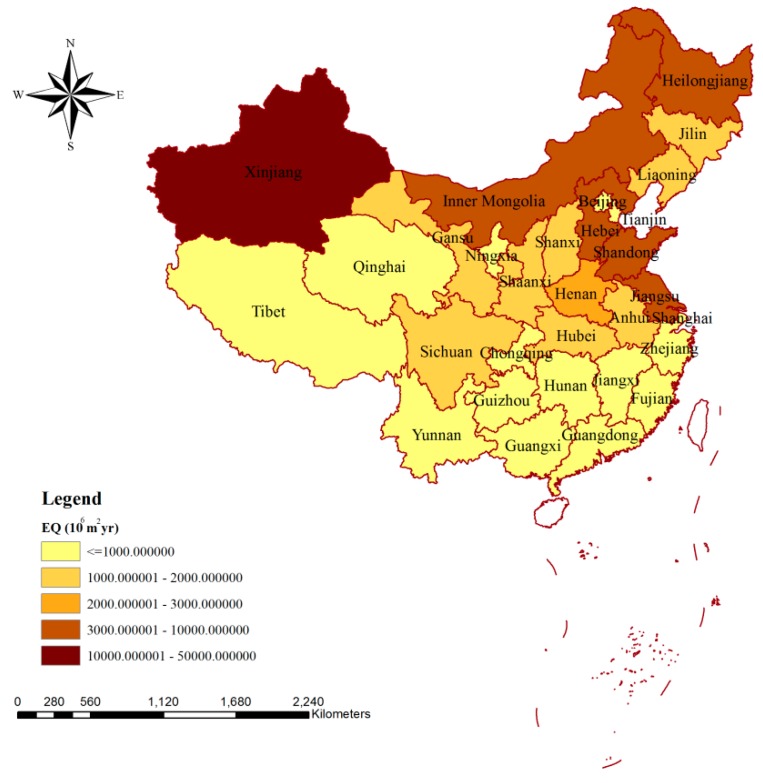
Spatial variation of the impacts of blue water consumption on ecosystem quality via production in China. Note: EQ means the impacts on ecosystem quality caused by production.

As can be seen from Figure 3, the ecosystem quality in Xinjiang was influenced most significantly by the blue water consumption caused by production behaviors, and the value of the influence was as high as 49.88 × 10^9^ m^2^∙year. For Shandong, Hebei, Heilongjiang, Jiangsu, and Inner Mongolia, the impacts on ecosystem quality were more than 3 × 10^9^ m^2^∙year. For half of the provinces of China, the impacts of blue water consumption during production on ecosystem quality were less than 1 × 10^9^ m^2^∙year, and the smallest influence could be observed in Tibet (189.35 × 10^6^ m^2^∙year), which was less than 1% of the value for Xinjiang.

**Figure 4:**
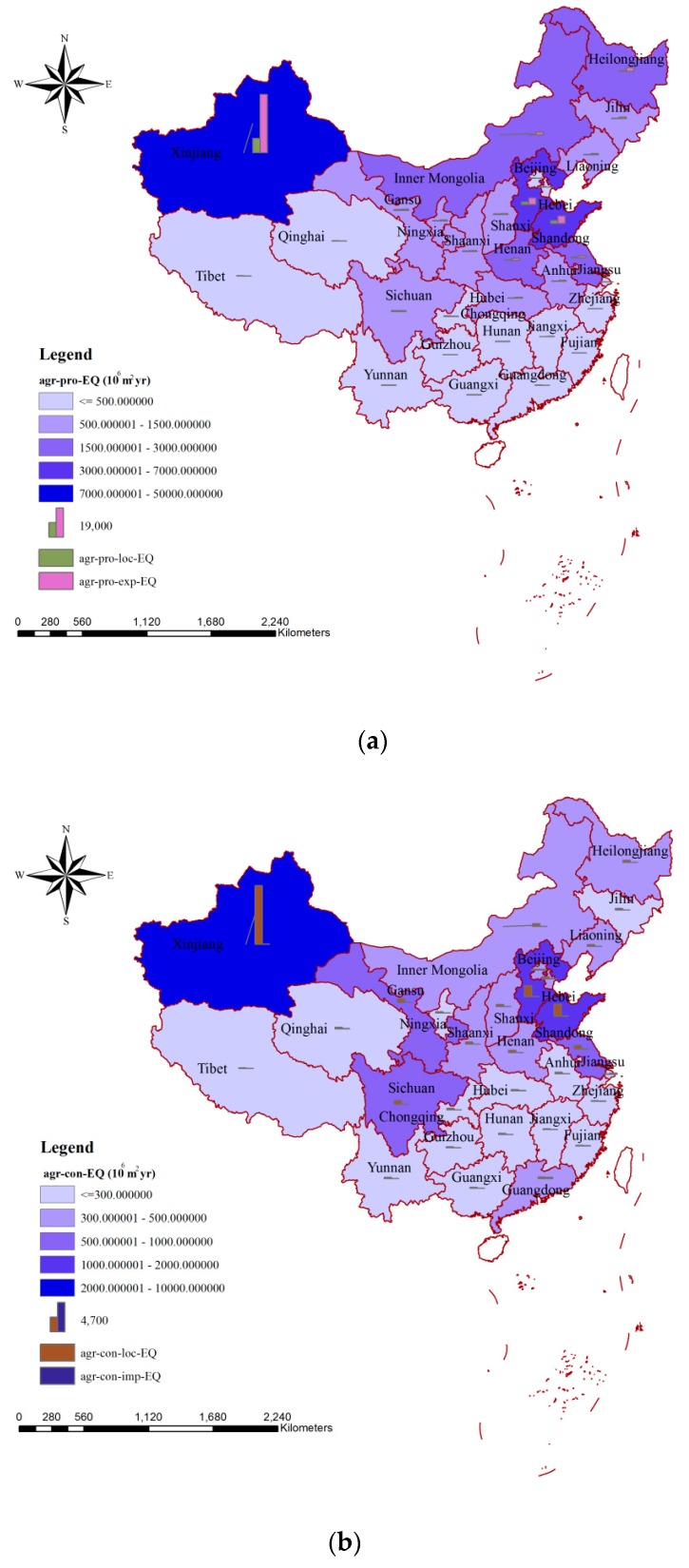
Spatial variation of the impacts of blue water consumption on ecosystem quality via agricultural production (**a**) and agricultural consumption (**b**) in China. Note: agr-pro-EQ, agr-pro-loc-EQ, and agr-pro-exp-EQ mean the impacts on ecosystem quality due to agricultural production, the part of agricultural production used for local consumption, and the part of agricultural production exported to meet the needs of inhabitants in other areas, respectively. agr-con-EQ, agr-con-loc-EQ, and agr-con-imp-EQ mean the impacts on ecosystem quality due to agricultural consumption, the part of agricultural consumption met by local production, and the part of agricultural consumption dependent on imports from other areas, respectively.

Figure 4 presents the spatial variation of the impacts of blue water consumption on ecosystem quality via agricultural production (Figure 4a) and consumption (Figure 4b), respectively. Xinjiang was the province with the largest impacts on ecosystem quality due to the blue water consumption for agricultural production (48.06 × 10^9^ m^2^∙year), and Shandong (6.99 × 10^9^ m^2^∙year) and Hebei (6.11 × 10^9^ m^2^∙year) took the second and third places, respectively (Figure 4a). A similar distribution could be seen for the impacts related to agricultural consumption, and the values for the top three were 9.40 × 10^9^ m^2^∙year (Xinjiang), 1.91 × 10^9^ m^2^∙year (Shandong), and 1.72 × 10^9^ m^2^∙year (Hebei) (Figure 4b). In terms of the smallest impacts on ecosystem quality, Shanghai and Tibet were the regions related to agricultural production and agricultural product consumption, respectively. For about two-thirds of the provinces in China, the impacts of blue water consumption on ecosystem quality via agricultural production were mainly related to exported agricultural products, and the largest impact due to agricultural export was 38.66 × 10^9^ m^2^∙year (Xinjiang) (Figure 4a). From the consumption perspective, the impacts on ecosystem quality were mainly due to the contribution from local production for most provinces, and the largest and smallest influences via agricultural consumption from local production were 9.40 × 10^9^ m^2^∙year (Xinjiang) and 41.36 × 10^6^ m^2^∙year (Shanghai) (Figure 4b).

### 3.3. Impacts on Resources

**Figure 5:**
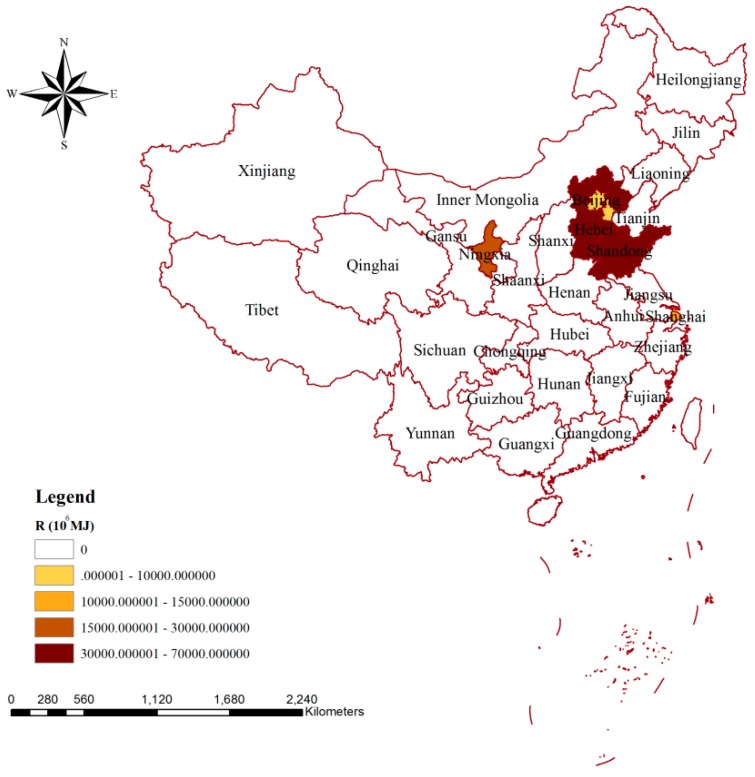
Spatial variation of the impacts of blue water consumption on resources via production in China. Note: R means the impacts on resources caused by production.

The impacts of blue water consumption on resources via production are shown in Figure 5. For Hebei and Shandong, the impacts on resources were more obvious than for other provinces, with their values being 62.21 × 10^9^ and 56.55 × 10^9^ MJ, respectively. There were two provinces (Ningxia and Shanghai) where the influence of blue water consumption during the production was between 10 × 10^9^ and 30 × 10^9^ MJ. For Tianjin and Beijing, the values of influences were less than 10 × 10^9^ MJ. For other provinces of China, the water withdrawals were less than the volume of local water availability, thus the water depletion could be compensated by precipitation, resulting in a value of zero for the impacts on water resources.

**Figure 6:**
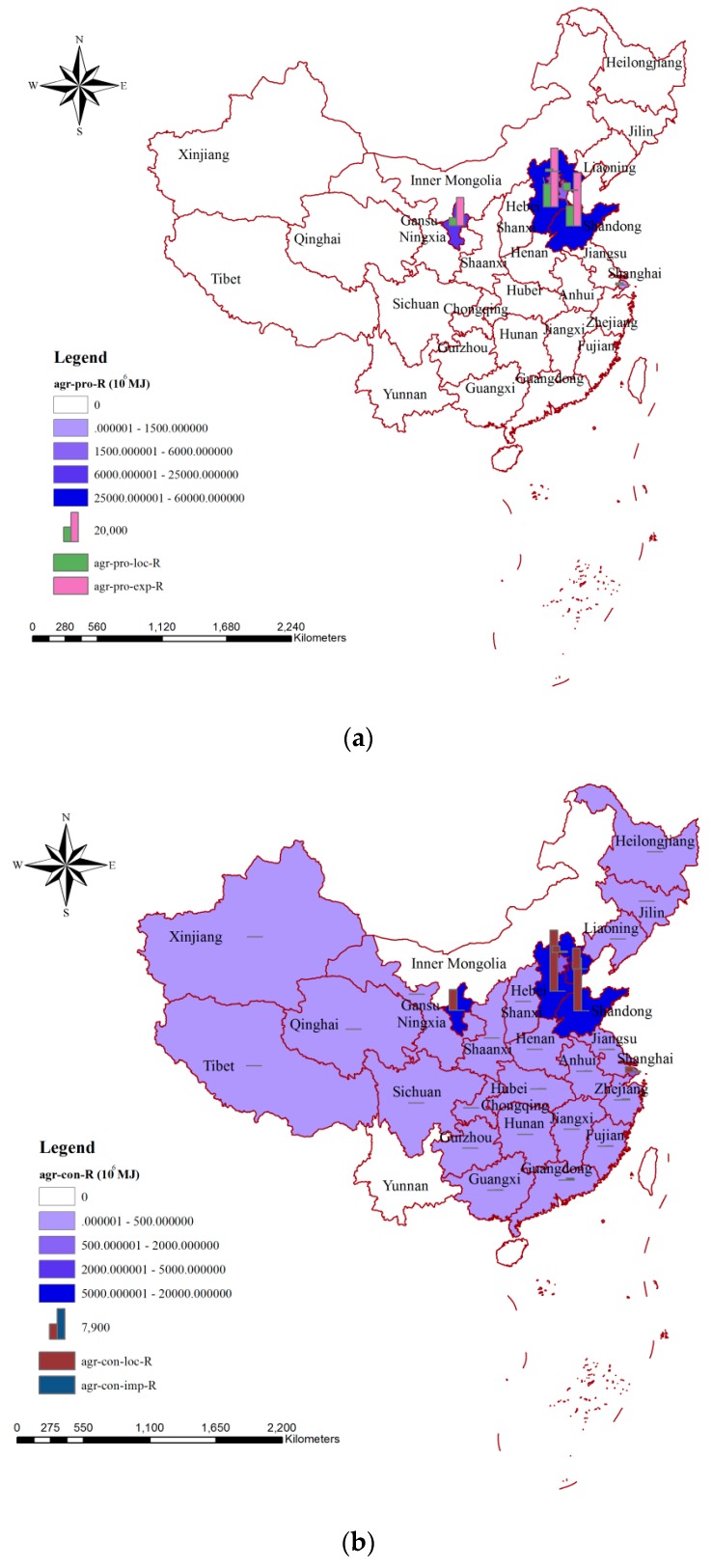
Spatial variation of the impacts of blue water consumption on resources via agricultural production (**a**) and agricultural consumption (**b**) in China. Note: agr-pro-R, agr-pro-loc-R, and agr-pro-exp-R mean the impacts on resources due to agricultural production, the part of agricultural production used for local consumption, and the part of agricultural production exported to meet the needs of inhabitants in other areas, respectively. agr-con-R, agr-con-loc-R, and agr-con-imp-R mean the impacts on resources due to agricultural consumption, the part of agricultural consumption met by local production, and the part of agricultural consumption dependent on importing from other areas, respectively.

A similar spatial distribution could be observed for the impacts of blue water consumption on resources via agricultural production in China (Figure 6a). The largest and smallest influences on water resources occurred in Hebei and Beijing, respectively, and the former was more than 33 times greater than that of the latter. Comparing the parts of agricultural production used locally and for export, we found that the impacts on resources in Hebei, Shangdong, and Ningxia were mainly related to agricultural production exported to other areas, while those impacts in Shanghai and Tianjin were mainly due to agricultural production used locally. For Beijing, all of the impacts on resources were due to agricultural production used locally. Figure 6b shows the spatial distribution of the impacts on resources related to agricultural consumption, and the values in Hebei (15.78 × 10^9^ MJ) and Shandong (13.76 × 10^9^ MJ) were much higher than those in other areas. There were 22 provinces where the impacts on water resources were less than 500 × 10^6^ MJ, and the values in Inner Mongolia and Yunnan were zero. For most of the provinces in China, the influences on water resources were dominated by the part of agricultural products that were imported, while for Shandong, Hebei, Ningxia, Tianjin, Beijing, and Shang, it was mainly related to agricultural products that were produced and consumed locally.

## 4. Discussion

Comparing the spatial distributions of the impacts of blue water consumption from production on human health, ecosystem quality, and resources, obvious differences could be observed (Figure 1, Figure 3 and Figure 5). This could be a result of the influences of regional blue water consumption or might be due to the impacts of various environmental factors. Table 1 shows the top five blue water consumers of China and the corresponding rankings for environmental influences. Xinjiang ranked first for both the impacts on human health and on ecosystem quality, which was mainly due to it having the largest blue water consumption. Although the blue water consumption of Henan occupied the third place for China, its environmental influences were much smaller than those of the provinces listed in Table 1, which was mainly due to the influences of various environmental factors. Taking the impacts on human health as an example, Henan was ranked 10th, 19th and 12th for WSI, ${\mathrm{WU}}_{\%,\mathrm{agr}}$, and HDF, respectively. Provinces such as Xinjiang and Henan could be targets in future for decreasing blue water consumption to achieve smaller environmental impacts. The agricultural sector was the largest contributor for the environmental influences in China, and more than 80% of the impacts on human health, ecosystem quality, and resources were due to the blue water consumption in agricultural production (Table 2). According to the study of Dalin and Rodríguez-Iturbe, agriculture will present increasingly harmful environmental impacts (including altering water, nitrogen, and carbon cycles and threatening health and landscape biodiversity) in the next decades, due to natural resource use and greenhouse gas emissions [25]. However, the government has neglected certain required maintenance for irrigation systems, and this was mainly due to the fact that most of the economic value of water was in industrial or residential sectors, rather than in the agricultural sector [55,56,57]. Thus, there is an urgent need to improve irrigation technologies and encourage water-saving practices, and the virtual water trades might also be adopted to achieve water saving at the national level [6,55].

Two perspectives were provided for the analysis of the environmental impacts in the agricultural sector. From the perspective of production, about 70% of the environmental impacts (74.60% on human health, 75.69% on ecosystem quality, and 69.16% on resources) were related to exported products (Figure 2a, Figure 4a and Figure 6a). From the consumption perspective, more than 95% of the environmental impacts were related to agricultural products produced locally, and the parts related to the importing of agricultural products were only 4.18%, 4.56%, and 4.53% for human health, ecosystem quality, and resources, respectively (Figure 2b, Figure 4b and Figure 6b). This was consistent with the fact that China is an important agricultural exporter and most of the agricultural products consumed by Chinese are from local production. China’s current export-oriented economic growth strategy—in the context of the hidden virtual flows of resources and environmental pressures—is not sustainable [58]. In cases where a region used high amounts of water for producing specific export products, a disregard of water stress and environmental impacts caused by this export-driven production would, in the medium-to-long run, result in irreversible damage to the water system and ecosystem in this region [59]. Thus, resource-scarce countries such as China must incorporate trade-off decisions between pursuing national economic growth, incurring environmental degradation, and achieving food security into strategic development policies, and related compensation for export should be considered in future [58]. On a global scale, approximately one billion people are chronically malnourished and the agricultural systems are concurrently degrading water, biodiversity, land, and others [60,61,62,63]. Thus, measures considering regional production and consumption characterizes should be taken to reduce the current environmental impacts. Water-intensive crops mean crops for which relatively large volumes of blue water are consumed in their agricultural production [19,64,65]. The shift from water-intensive crops to less water-intensive ones could generate a decrease in blue water consumption and increase water saving for production regions and have smaller environmental impacts based on the methods we mentioned above. In terms of production, measures such as the shift of the agricultural production pattern from less water-intensive crops and animal products to water-intensive ones; the increase of agricultural water use efficiency, especially that for irrigation; and the adoption of water-saving technologies could contribute to producing smaller environmental impacts [17,66,67]. The largest impact both on human health and on ecosystem quality could be seen in Xinjiang, while the agricultural irrigation efficiency in this province was only 0.527. The value was much lower than in Beijing (0.710) or Shanghai (0.735), where the agricultural irrigation systems are more advanced. If the irrigation efficiency in the Xinjiang province could be increased to the level in Shanghai, about 9.69 × 10^9^ m^3^ of water resources could be saved for the agricultural sector, and the corresponding impacts on human health and ecosystem quality would be decreased by 1.87 × 10^6^ DALY and 13.60 × 10^9^ m^2^∙year, respectively. In terms of consumption, besides improving local agricultural production practices, the main focus should be on increasing awareness of the importance of saving water and whether products are imported from regions with relatively small environmental impacts [59,68]. Both Hebei and Shandong are important agricultural product producing and exporting regions, but their blue water consumption and environmental impacts by mass of product are significantly different. Taking maize as an example, if 1 kg of maize was imported from the Hebei province, 0.139 m^3^ of blue water would be consumed and the related environmental impacts would be 1.786 × 10^−5^ DALY on human health, 0.046 m^2^∙year on ecosystem quality, and 0.426 MJ on resources, whereas if the 1 kg of maize was imported from Shandong, only 0.061 m^3^ of blue water would be needed and the related environmental impacts, which would be 5.333 × 10^−5^ DALY, 0.019 m^2^∙year, and 0.140 MJ, respectively, would be much smaller than those in Hebei. Hu et al. analyzed the environmental impacts due to water consumption for the main agricultural products in Lake Dianchi Basin (located in the Yunan province of China), and they found that to produce 1 kg of rice, bovine meat, and swine meat, the corresponding environmental impacts on ecosystem quality were 0.060, 0.114, and 0.107 m^2^∙year, respectively [69]. In their study, both blue and grey water were considered. According to our study, the corresponding environmental impacts on ecosystem quality were 0.024, 0.046, and 0.038 m^2^∙year, respectively, according to the blue water consumption required to produce 1 kg of rice, bovine meat, and swine meat. If both blue and grey water were considered, the impacts on ecosystem quality would be 0.052, 0.084, and 0.100 m^2^∙year, respectively [30,31]. Consequently, the percentage differences between our study and that of Hu et al. were 15.41% (rice), 36.36% (bovine meat), and 6.57% (swine meat) respectively.

Compared with traditional LCA studies, which mainly focus on land and soil use, energy use, and material consumption [2,20,21], this study analyzed the environmental impacts of blue water consumption, with the help of the water footprint concept. In this study, only the agricultural sector was considered for the evaluation of environmental impacts from the consumption perspective, which was mainly due to the lack of data for the industrial and other sectors. In future research, an evaluation conducted for all sectors would provide a more complete picture. Besides the environmental impacts, the social or economic impacts should also be included in our future study to improve regional water management in a broader context [12,70]. Furthermore, malnutrition, reduced biodiversity, and increased extraction costs are only some of many possible effects. In our study, damage arising from reduced water availability for hygiene depended on local circumstances, e.g., the distance between the residents and the nearest well, and was therefore difficult to assess [44]. Last but not least, green water is crucial to support plant growth in rain-fed regions, and a comparison of different models has shown that green water consumption for agricultural production was about 4 or 5 times greater than blue water consumption in global crop production [71,72,73]. Studies focused on the environmental impacts of green water consumption have been conducted in recent years. Nunez et al. assessed the environmental impact of green water consumption for four typical crop rotations grown in Spain by accounting for the difference of green water demand in an actual and a reference situation and putting forward a green-water-scarcity index [74]. Quinteiro et al. proposed a method of assessing impacts on terrestrial green water flows, taking into account the green water use/atmosphere and green water use/soil interfaces [75]. Quinteiro et al. developed two new sets of spatially differentiated and globally applicable characterization factors to assess the environmental impact of green water flows [73]. The focus of this manuscript was blue water resources, and the environmental impacts of green water consumption were not included, which was mainly due to the lack of data. In future, a comprehensive analysis for different kinds of water resources should be conducted to improve regional water management.

## 5. Conclusions

In this study, the impacts on human health, ecosystem quality, and water resources in China due to blue water consumption were evaluated from the production and consumption perspectives, respectively, with the help of the water footprint concept. The following conclusions could be drawn: The environmental impacts due to blue water consumption of different sectors in China were 15.82 × 10^6^ DALY, 96.54 × 10^9^ m^2^∙year, and 175.20 × 10^9^ MJ, respectively, and provinces such as Xinjiang, Shandong, and Hebei could be future targets for decreasing blue water consumption to obtain smaller environmental impacts. More than 80% of environmental impacts were related to the agricultural sector.

In terms of agricultural production, about 70% of the environmental impacts were related to exported products. China’s current export-oriented economic growth strategy is not sustainable, thus resource-scarce countries such as China must incorporate trade-off decisions between pursuing national economic growth, incurring environmental degradation, and achieving food security into strategic development policies. Measures such as the shift of agricultural production pattern from water-intensive crops and animal products toward less water-intensive ones; the increase of agricultural water use efficiency, especially for irrigation; and the adoption of water-saving technologies could contribute to smaller environmental impacts.

In terms of agricultural consumption, more than 95% of the environmental impacts were related to agricultural products produced locally. The main focus should be on increasing awareness of the importance of water saving and whether products are imported from regions with relatively small environmental impacts.

## Figures and Tables

**Table 1:** The top five blue water consumers and the corresponding rankings for environmental influences.

Rank	Province	Ranking for Impacts on Human Health	Ranking for Impacts on Ecosystem Equality	Ranking for Impacts on Resources
1	Xinjiang	1	1	7
2	Shandong	3	2	2
3	Henan	14	7	7
4	Hebei	2	3	1
5	Jiangsu	6	5	7

Note: the rankings for impacts on resources were ranged from 1 to 7 and the value of 7 means that the impacts of blue water consumption via production were zero.

**Table 2:** The environmental influences caused by blue water consumption from production in different sectors of China.

	Agriculture (%)	Industry (%)	Domestic (%)	Artificial Ecosystem (%)
Impacts on human health	88.93	3.60	4.97	2.50
Impacts on ecosystem equality	85.93	5.11	6.21	2.75
Impacts on resources	80.15	6.52	8.46	4.87
